# Anaesthetic management in patients with glucose-6-phosphate dehydrogenase deficiency undergoing neurosurgical procedures

**DOI:** 10.4103/0019-5049.76597

**Published:** 2011

**Authors:** Sebastian Valiaveedan, Charu Mahajan, Girija P Rath, Ashish Bindra, Manish K Marda

**Affiliations:** Department of Neuroanaesthesiology, All India Institute of Medical Sciences, New Delhi, India

**Keywords:** Glucose-6-phosphate dehydrogenase (G-6-PD) deficiency, haemolysis, neurosurgery, oxidative stress

## Abstract

Glucose-6-phosphate dehydrogenase (G-6-PD) deficiency is an X-linked recessive enzymopathy responsible for acute haemolysis following exposure to oxidative stress. Drugs which induce haemolysis in these patients are often used in anaesthesia and perioperative pain management. Neurosurgery and few drugs routinely used during these procedures are known to cause stress situations. Associated infection and certain foodstuffs are also responsible for oxidative stress. Here, we present two patients with G-6-PD deficiency who underwent uneventful neurosurgical procedures. The anaesthetic management in such patients should focus on avoiding the drugs implicated in haemolysis, reducing the surgical stress with adequate analgesia, and monitoring for and treating the haemolysis, should it occur.

## INTRODUCTION

Glucose-6-phosphate dehydrogenase (G-6-PD) deficiency is an X-linked recessive genetic disease, caused by deficiency of an enzyme in the hexose-mono-phosphate (HMP) shunt pathway of carbohydrate metabolism, and results in decreased production of nicotinamide adenine dinucleotide phosphate hydrogenase (NADPH). NADPH protects cells from oxidative stress, thus, defective red blood cells (RBC) are more susceptible to haemolysis by oxidative stress.[1] The condition presents 1-3 days following the oxidative stress and the resultant haemolysis usually resolves, uneventfully. It is a benign condition and adoption of preventive measures can ensure safe administration of anaesthesia. Our search of literature did not reveal any G-6-PD-deficient patient being anaesthetised for neurosurgical procedures.

## CASE REPORTS

### Case 1

A 2-year-old female child with G-6-PD deficiency, weighing 13 kg was admitted to our neurosurgical unit with the complaints of a swelling over the lower back and weakness of both the lower limbs. An magnetic resonance imaging (MRI) of spine revealed lumbosacral lipoma with tethered cord syndrome. Surgical excision of the lipoma with release of cord was planned. There was no history haemolysis, jaundice or blood transfusion. All the routine investigations were within normal limits (haemoglobin 13.6 g%, unconjugated bilirubin 0.7 mg%, and reticulocyte count 1%). The child was premedicated with oral atropine and midazolam. In the operating room, inhalational induction with sevoflurane in oxygen was carried out and an intravenous (IV) access was secured, simultaneously. Fentanyl 2 *μ*g/kg was administered and tracheal intubation was facilitated with rocuronium 1mg/kg. The child was placed prone for surgery. Intraoperative monitoring included electrocardiogram (ECG), oxygen saturation (SpO_2_), non invasive blood pressure (NIBP) and end tidal carbon dioxide (EtCO_2_), temperature. Anaesthesia was maintained with sevoflurane, O_2_/N_2_O mixture and intermittent boluses of rocuronium and fentanyl. Cloxacillin 200 mg and amikacin100 mg were given IV as prophylactic antibiotics. The blood loss was 150 ml, urine output was 90 ml, and 650 ml of Ringers lactate was infused. The uneventful surgery lasted for 4 h. At the end of procedure, the child was placed supine, anaesthetic agents discontinued. Rectal paracetamol 500 mg suppository was given for postoperative analgesia. Residual neuromuscular blockade was reversed with neostigmine and glycopyrrolate and trachea was extubated. On third postoperative day (POD), the child was discharged after ensuring the complete blood count (CBC) to be normal.

### Case 2

A 36-year-old male patient, weighing 61 kg, presented with a history of episodic headache for 10 years and decreased vision in the left eye for 6 months. He was diagnosed with G-6-PD deficiency and had no history of haemolytic episodes or blood transfusion. Computed tomographic (CT) scan of head revealed a planum sphenoidale meningioma. All other routine investigations were within normal limits. Craniotomy and excision of the tumour was planned. The patient was started on mannitol, dexamethasone and glycerol to reduce intracranial pressure and decrease brain oedema. After premedication with intramuscular glycopyrrolate 0.2 mg, anaesthesia was induced with propofol 150 mg and fentanyl 150 *μ*g. Tracheal intubation was facilitated with rocuronium 60 mg. Anaesthesia was maintained with isoflurane, O_2_/N_2_O mixture, rocuronium and fentanyl. Cloxacillin and amikacin 500 mg each were given as prophylactic antibiotics. The surgery was performed in the supine position, lasted for 7 h. Monitoring modalities included ECG, SpO_2_, EtCO_2_, temperature, arterial blood pressure (BP), central venous pressure (CVP) and urine output. Intraoperatively, the patient received 300 ml of mannitol, 4600 ml of crystalloid (Ringer’s lactate 1000 ml and normal saline 3600 ml) and 500 ml of 6% hydroxyethyl starch. The total blood loss was 600 ml. At the end of surgery, the patient was transferred to intensive care unit (ICU) for overnight ventilation, in view of inadequate haemostasis and hypothermia following prolonged surgery. Fentanyl 1-2 *μ*g/kg/h was infused for postoperative sedation and analgesia. The trachea was extubated, next day. On the third POD, the CBC was found to be normal and he was discharged on fourth POD.

## DISCUSSION

Various drugs, foods (favism) and infections are known to cause oxidative stress, thereby, haemolysis in G-6-PD-deficient patients. Hence, acute haemolytic anaemia is a frequent clinical manifestation.[1] Acute intravascular haemolysis starts 2-3 days following exposure. Recovery is marked by reticulocytosis. Laboratory findings following a haemolytic episode may include anaemia, reticulocytosis, decreased serum haptoglobulin and an elevated indirect bilirubin. Specific tests include enzyme levels in RBCs and Heinz body preparations. The diagnostic test for G-6-PD deficiency is a fluorescent spot test. In our patients, CBC was estimated in the postoperative period to rule out haemolytic episodes. As the clinical signs of haemolytic anaemia were absent, additional tests were not carried out.

Anaesthetic management should focus on avoiding the drugs implicated in haemolysis, and monitoring for and treating the haemolysis, should it occur.[2] Haemolysis is influenced by the type of mutation causing disease, genetic make-up and gender of the individual, age of erythrocyte and the type and dose of offending drug.[3] Petz and others.[4] listed a group of drugs, known to cause immune haemolytic anaemia. Most such drugs are used in the perioperative period include antimicrobials (sulfonamides, nitrofurantoin and chloramphenicol), non sterioidal antiinflamatory drugs (NSAIDs), anticonvulsants, diuretics, insulin, ranitidine and thiopentone.[5] The mechanism of immune haemolysis is distinctly different from that seen in G-6-PD deficiency which occurs due to membrane damage by oxidized haemoglobin. The fact that these agents can trigger haemolysis should be borne in mind while administering them, and dose limit should be strictly adhered. In this case, the antibiotics used are safe in G-6-PD-deficient patients.

Thiopentone and phenytoin are the two drugs deserve special mention in the context of neurosurgery. The former is an invaluable agent in neuroanaesthesia practice, while the later is routinely administered for seizure prophylaxis. Titrated administration of phenytoin with maintenance of safe plasma levels is necessary. However, there is wide inter-patient variability in serum phenytoin levels with equivalent doses. The liver enzyme system hydroxylating phenytoin is saturable at higher plasma levels. Hence, incremental doses of even 10% may produce toxicity, warrant extra caution during the administration. Thus, the safety of using higher doses of these agents in patients presenting for neurosurgery in the setting of G-6-PD deficiency needs to be established. Mannitol has antioxidant and free radical scavenging properties; hence, it is safe in G-6-PD-deficient patients. Mannitol was used in the second patient to reduce the intracranial pressure along with moderate hyperventilation. Generally, all anaesthetic agents are considered safe in G-6-PD deficiency and none of the currently used agents have been implicated; our experience supports this view. Agents like isoflurane and sevoflurane (for 4-8 h), fentanyl (5-10 *μ*g/kg), and rocuronium were used in these patients, without any adverse effect.

Reperfusion following ischaemia leads to increased vascular permeability and production of oxidative radicals by neutrophils and inflammatory cells. Central nervous system (CNS) ischaemia and reperfusion is a commonly encountered situation in neurosurgery. The ability of tissues to cope with oxidative stress posed by reperfusion following ischaemia may be compromised in the G-6-PD-deficient patients. The report of occurrence of re-expansion pulmonary oedema in a G-6-PD-deficient individual following thoracic sympathectomy[6] points to the existence of such a possibility. During active infections, the oxygen free radicals are produced either by inflammatory neutrophils or by use of antimicrobials may precipitate haemolysis during the perioperative period. This underlines the importance of adequate treatment of active infections before surgery in such patients.

Stress related to surgery per se causing haemolysis has been reported.[2] Hence, it is advisable to provide liberal analgesia and anxiolysis in the perioperative period. Enzyme (G-6-PD) levels are also low in platelets. However, *in vivo/vitro* studies showed no deleterious effect on platelet function.[7] Hence, coagulation parameters are normal in these patients. We avoided use of drugs implicated for inducing oxidative stress, and provided adequate analgesia/anxiolysis in the perioperative period that contributed to an uneventful anaesthetic course.

Acute haemolytic crisis in the intraoperative period should be strictly watched for as the immediate signs are typically masked during anaesthesia. Hypotension is a non-specific indicator of the crisis, may not be identified till haematuria is noticed. Once diagnosed, discontinuation of the offending agent and maintenance of urine output by infusion of crystalloids along with diuretics has to be ensured. Postoperatively, CBC on daily basis should be followed to monitor the need for blood transfusion.

To conclude, though essentially a benign condition, G-6-PD deficiency poses certain unresolved issues in view of current hypotheses relating to drug-induced haemolysis and mechanisms of injuries to the nervous system. How these will affect perioperative management and patient outcome in patients presenting for surgery, with special reference to neurosurgical procedures remains to be unraveled.
